# Bridging the Gap Between Awareness and Practice in Breast Cancer Screening: A Cross-Sectional Study

**DOI:** 10.3390/healthcare14131981

**Published:** 2026-07-03

**Authors:** Dimitra Georga, Maria Saridi, Erasmia Rouka, Dimitra Latsou, Evangelos C. Fradelos, Constantinos Togas, Pavlos Sarafis, Ioanna V. Papathanasiou, Dimitrios Papagiannis, Aikaterini Toska

**Affiliations:** 1 Department of Nursing, University of Thessaly, 41500 Larissa, Greece; dimig17@yahoo.gr (D.G.); msaridi@uth.gr (M.S.); errouka@uth.gr (E.R.); efradelos@uth.gr (E.C.F.); psarafis@uth.gr (P.S.); iopapathanasiou@uth.gr (I.V.P.); dpapajohn@uth.gr (D.P.); ktoska@uth.gr (A.T.); 2 Department of Business Administration, Sector of Social Policy, University of West Attica, 12241 Athens, Greece; demilatsou@yahoo.gr; 3 Department of Educational Sciences and Social Wok, University of Patras, 26504 Patra, Greece

**Keywords:** breast cancer screening, mammography, breast self-examination, knowledge, attitudes, preventive behavior, public health

## Abstract

Background/Objectives: Breast cancer is the most prevalent malignancy among women worldwide and a major public health concern. Early detection through screening methods such as breast self-examination, clinical breast examination, and mammography is associated with improved prognosis and reduced mortality. However, adherence to screening recommendations remains suboptimal and is influenced by multiple sociodemographic and psychosocial factors. Aims: This study aimed to assess women’s knowledge, attitudes, and practices regarding breast cancer screening and to identify factors associated with preventive behavior. Methods: A cross-sectional study was conducted using a structured self-administered questionnaire. The sample consisted of 1233 women from the general population. Data collected included sociodemographic characteristics, awareness of screening methods, attitudes toward prevention, and screening practices. Statistical analysis involved descriptive measures and multivariate regression models to identify predictors of knowledge and attitudes. Results: High levels of awareness were observed, with 96.5% reporting knowledge of breast self-examination, 94.5% clinical examination, and 95.0% mammography. Despite this, a gap between knowledge and practice was evident. Although 79.5% reported knowing how to perform breast self-examination, only 22.7% practiced it monthly, and 19.2% never performed it. Clinical examination within the past year was reported by 49.4%, while 13.3% had never undergone it. Among the women 45+ years old, 86% reported undergoing mammography every two years. Education, income, age, and place of residence were significantly associated with outcomes. Conclusions: A substantial gap exists between awareness and practice. Effective interventions should address behavioral, psychosocial, and structural barriers to improve screening uptake.

## 1. Introduction

Breast cancer is the most common malignancy in women worldwide and a significant cause of mortality, constituting a major public health issue [1]. Early diagnosis through screening has been shown to contribute significantly to reducing mortality and improving survival. The main screening methods include breast self-examination, clinical breast examination, and mammography, with mammography being the main method of population screening [2].

Despite the documented effectiveness of these methods, women’s compliance with screening recommendations remains inadequate. Screening participation is influenced by a multifactorial system of determinants, including sociodemographic, economic, and geographical variables [3,4]. Age, educational level, income, and place of residence have been repeatedly associated with variations in the use of preventive services, with lower participation rates observed in populations of lower socioeconomic status and in rural areas [5,6].

In addition, psychosocial factors, such as health beliefs, perceived vulnerability, fear of diagnosis, and self-efficacy, have been identified as critical determinants of preventive behavior [7,8]. Of particular importance is the documented gap between knowledge and behavior: despite relatively high levels of awareness, the adoption of preventive practices remains limited [9,10]. This finding suggests that knowledge, although necessary, is not a sufficient condition for behavioral change. In the Greek population, the utilization of screening services presents significant inequalities, despite the recent development of organized prevention programs. The implementation of national initiatives, such as the “Fofi Gennimata” program, aims to improve access and increase participation; however, socioeconomic and geographical inequalities remain (European Observatory on Health Systems and Policies [11]. At the same time, qualitative studies in the Greek area have highlighted that factors such as cultural perceptions, fear, and practical barriers significantly influence participation in preventive screening [12,13]. Considering the above, investigating women’s knowledge, attitudes, and practices regarding breast cancer screening becomes particularly important. Understanding the determinants of preventive behavior can contribute to the design of targeted and effective public health interventions.

The purpose of this study was to investigate women’s attitudes, knowledge, and practices regarding breast cancer screening, as well as to identify factors that influence their participation in preventive practices.

## 2. Materials and Methods

### 2.1. Study Design

A quantitative cross-sectional study was conducted to assess women’s knowledge, attitudes, and beliefs regarding breast cancer screening in Greece. Data collection was carried out nationwide between June 2024 and December 2025. The extended recruitment period was necessary due to the nationwide scope and large sample size of the study.

### 2.2. Sample and Sampling Procedure

The study sample consisted of 1233 women aged 21–69 years who were residents of Greece. A non-probability snowball sampling technique was employed. Participants were initially recruited through the researchers’ professional and social networks and via social media platforms and were subsequently encouraged to share the questionnaire with other eligible women. This approach facilitated the recruitment of participants from different geographical regions of Greece; however, it may have introduced selection bias and limited the representativeness of the sample. Participants were recruited through digital platforms and printed materials, allowing the inclusion of women from diverse geographical regions and socioeconomic backgrounds.

Eligible participants were women aged 21–74 years residing in Greece. This age range was selected to support a comprehensive preventive approach, encompassing both early awareness and participation in organized screening programs. The inclusion of younger women (≥21 years) is consistent with international guidelines emphasizing early awareness and symptom recognition, as outlined by the World Health Organization [1] and the American Cancer Society [14]. Moreover, the study aligns with the Greek National Breast Cancer Screening Program “Prolamvano (I PREVENT)–Fofi Gennimata,” which targets women between 45 to 74 years old, thereby enhancing the policy relevance of the findings [11]. The selected age range also ensured compatibility with the validated Breast Cancer Screening Beliefs Questionnaire (BCSBQ), supporting the reliability of the data collected through digital means.

According to the 2021 population census of the Hellenic Statistical Authority, the number of women aged 21 to 74 years in Greece was 3,651,891. The required sample size was calculated using the Raosoft sample size calculator (Raosoft Inc., Seattle, WA, USA), with a 3% margin of error, a 95% confidence level, and a 50% response distribution. Based on these parameters, the minimum required sample size for the present study was estimated at 1068 participants. The final study sample consisted of 1233 complete questionnaires, exceeding the minimum required sample size and strengthening the adequacy of the sample for supporting the nationwide scope of the study.

A non-probability snowball sampling technique was employed. Participants were initially recruited through the researchers’ professional and social networks and via social media platforms and were subsequently encouraged to share the questionnaire with other eligible women. This approach facilitated the recruitment of participants from different geographical regions of Greece. To minimize the exclusion of women with limited digital literacy, the questionnaire was also made available in print and administered to women who were unfamiliar with or unable to use digital technologies. The combined use of online and paper-based questionnaires aimed to increase inclusiveness and facilitate the participation of women from diverse geographical regions and socioeconomic backgrounds. The questionnaire was self-administered and completed anonymously by eligible participants. All returned questionnaires were checked for completeness before statistical analysis. Questionnaires with substantial missing information or incomplete responses in the main study variables were excluded from the final dataset.

It is important to mention that although women aged 21–74 years were invited to participate in the study, no completed questionnaires were received from women aged 70–74 years, either electronically or in paper format. Therefore, the final analytical sample included women aged 21–69 years. This should be acknowledged as a limitation, as the views and screening-related beliefs of women in the oldest eligible age group were not represented in the present study.

The questionnaire was self-administered and completed anonymously by eligible participants. All returned questionnaires were checked for completeness before statistical analysis. Questionnaires with substantial missing information or incomplete responses in the main study variables were excluded from the final dataset. For the scale-based variables, total or subscale scores were calculated according to the scoring instructions of each instrument. Responses such as “I do not know/I do not answer”, where provided as an explicit response option, were treated as valid categorical responses and not as missing data. No statistical imputation of missing values was performed. For regression analyses, cases with missing values in the dependent variable or in any of the independent variables included in each model were excluded using listwise deletion.

### 2.3. Study Instrument

Data were collected using the Breast Cancer Screening Beliefs Questionnaire (BCSBQ) (Toska et al., 2025) [15]. The BCSBQ is a validated instrument designed to assess women’s beliefs and attitudes toward breast cancer screening, particularly in culturally diverse populations. It typically comprises multiple subscales that evaluate domains such as knowledge and perceptions of breast cancer, attitudes toward screening practices (e.g., mammography), and perceived barriers to participation in screening programs.

Responses are recorded using a Likert-type scale, allowing for the quantification of participants’ beliefs and facilitating comparative analysis across subgroups. Higher scores indicated more positive attitudes, greater knowledge, or lower perceived barriers. The instrument has demonstrated satisfactory psychometric properties, including internal consistency and construct validity, in previous studies [16,17].

### 2.4. Ethical Considerations

The study was conducted in accordance with the principles of research involving human participants and the Declaration of Helsinki. Ethical approval was obtained before the initiation of the study from the Department of Nursing, University of Thessaly Ethics and Deontology Committee (approval number: 391/12-07-2024). All participants were informed about the purpose of the study and provided informed consent before participation. Participation was voluntary and anonymous, and data confidentiality was strictly maintained.

### 2.5. Statistical Analysis

All statistical analyses were performed using IBM SPSS Statistics for Windows, version 25.0 (IBM Corp., Armonk, NY, USA). Descriptive statistics were calculated for all study variables. Categorical variables are presented as frequencies and percentages, while continuous variables are reported as means and standard deviations. The normality of continuous variables was evaluated using the Kolmogorov–Smirnov test. One-way analysis of variance (ANOVA) was used to examine differences in the Breast Cancer Screening Beliefs subscales according to sociodemographic and reproductive characteristics. Pearson’s chi-square test was applied to assess associations between categorical variables in the crosstabulation analyses. Backward linear regression analysis was conducted to identify significant sociodemographic and reproductive predictors of the three subscales. A *p*-value of less than 0.05 was considered statistically significant.

For the scale-based variables, total or subscale scores were calculated according to the scoring instructions of each instrument. Responses such as “I do not know/I do not answer”, where provided as an explicit response option, were treated as valid categorical responses and not as missing data. No statistical imputation of missing values was performed. For regression analyses, cases with missing values in the dependent variable or in any of the independent variables included in each model were excluded using listwise deletion.

## 3. Results

### Demographic Characteristics

In Table 1, the demographic characteristics are presented. The largest age group was 45–54 years (26.1%), followed by those aged 35–44 years (23.4%). Regarding family status, 63.9% were married. In terms of educational level, nearly half of the participants completed tertiary education (49.2%), while 23.6% had postgraduate studies. The majority resided in urban areas (76.9%), and most were employed (72.8%). Regarding monthly family income, the largest proportion reported an income of more than €1501 (36.4%), followed by €1001–€1500 (29.0%).

Concerning smoking-related characteristics, 67.9% of participants reported being smokers, and 56.2% reported exposure to passive smoking.

Concerning reproductive history (Table 2), most participants reported having two children (41.1%) and two pregnancies (32.6%). In terms of mode of delivery, most women reported no history of normal childbirth (59.8%), whereas 19.8% had undergone two normal deliveries. Similarly, 66.6% of participants reported no history of caesarean section, while 15.9% had undergone two caesarean sections and 14.9% had undergone one. Regarding pregnancy loss, more than half of the participants (56.8%) reported no history of miscarriage or abortion, whereas 25.0% had experienced one. Concerning assisted reproduction, the vast majority (92.5%) reported no history of IVF. Among those with IVF history, most had undergone one cycle (38%).

Most participants reported no personal history of breast cancer. Concerning the Breast Cancer Screening Beliefs Subscale, the findings suggest that the sample demonstrated more proactive attitudes (mean = 79.5 ± 23.5), higher levels of knowledge (mean = 79.3 ± 21.2), and lower perceived barriers to mammography (mean = 89.4 ± 15.4).

Table 3 presents the breast screening practices. Regarding family and social history, 20.1% of participants reported a family history of breast cancer. Similarly, 42.1% reported a history of breast cancer among friends. Awareness of breast self-examination was very high, with 96.5% of participants reporting that they were familiar with breast self-examination; however, a smaller proportion (79.5%) stated that they knew how to perform it, suggesting a gap between general awareness and practical knowledge. In terms of practice, 36.0% of women reported performing breast self-examination once every few months, and 19.2% never. Because the study included younger women who were not necessarily within the age groups targeted by organized mammography screening, these frequencies should not be interpreted as adherence rates to national screening recommendations. The main reasons were due to negligence, that they do not know how to do it, or that they prefer to have the examination performed by a doctor.

Knowledge of clinical breast examination was also high, as 94.5% of women reported being aware of it. Nearly half (49.4%) had undergone a clinical breast examination within the past year, while 13.3% had never undergone such an examination because they are young (<30 years). As for the reason for the most recent clinical breast examination, most women stated that they decided independently to have it as part of a health check-up (56.2%) (Table 3).

Awareness of mammography was likewise high (95.0%). In Greece, mammography refers to women over 45 years old. 86% of these women reported undergoing mammography every two years. The main reasons for undergoing mammography were early detection and participation in a routine health check-up. Most participants (60.2%) reported no preference regarding the gender of the mammography operator. Finally, 44.2% of participants reported that their friends undergo mammography, 21.2% reported that they do not, and 34.6% stated that they did not know (Table 3).

Table 4 presents significant differences among sociodemographic and reproductive characteristics and Breast Cancer Screening Beliefs Subscale.

The subscale attitudes toward general health check-ups differed significantly across age groups (F = 4.123, *p* = 0.007), with the highest mean score observed among women aged 25–34 years (mean = 83.2) and the lowest among those aged 65+ years (mean = 68.8). Significant differences in attitudes were also found according to educational level (F = 10.928, *p* < 0.001), with attitudes increasing progressively across levels of education and reaching the highest mean among women with postgraduate studies (mean = 83.40). Place of residence was also associated with attitudes (F = 12.128, *p* = 0.001), as women living in urban areas reported higher attitude scores than those living in rural areas (80.88 vs. 75.17). In addition, monthly family income was likewise significantly associated with attitudes (F = 9.297, *p* < 0.001), with the highest scores observed among women with a monthly income above €1501 (mean = 84.49). A statistically significant difference was also found according to the number of children (F = 2.688, *p* = 0.045), with the highest mean attitude score reported among women with two children (mean = 81.54).

Regarding subscale knowledge and perceptions about breast cancer, statistically significant differences were identified according to age (F = 6.738, *p* < 0.003), educational level (F = 13.474, *p* < 0.001), place of residence (F = 11.958, *p* = 0.001), family income (F = 4.341, *p* = 0.002), number of children (F = 3.774, *p* = 0.010), number of pregnancies (F = 3.437, *p* = 0.016), and history of normal childbirth (F = 8.446, *p* < 0.001). Younger participants demonstrated higher knowledge scores, particularly those aged 21–24 years (mean = 84.5), whereas the lowest score was observed in the 65+ age group (mean = 70.4). Knowledge scores also increased with educational level, ranging from 60.16 among women with primary education to 82.17 among those with postgraduate studies. Women living in urban areas reported higher knowledge scores than those in rural areas (80.41 vs. 75.29). In terms of family income, as income increases, so does the knowledge score. Furthermore, women without children had the highest knowledge score (mean = 82.20), whereas lower scores were observed among women with two or more children. A similar pattern was found for pregnancy history, with women reporting no pregnancies showing the highest knowledge score (mean = 81.72), compared with lower scores among those with more than two pregnancies (mean = 75.73). Finally, knowledge differed according to normal childbirth history (F = 8.446, *p* < 0.001), with the highest score among women with no history of normal childbirth (mean = 81.35) and the lowest among those with more than two normal deliveries (mean = 72.50).

For subscale barriers to mammographic screening, statistically significant differences were observed only according to place of residence (F = 4.124, *p* = 0.043), with women residing in urban areas reporting slightly lower barrier scores than those living in rural areas (89.89 vs. 87.69). No other statistically significant differences in barriers were identified across the remaining sociodemographic or reproductive characteristics examined.

The crosstabulation analysis showed that awareness of breast self-examination was significantly associated with both age and educational level (Table 4). Specifically, a statistically significant association was found between age group and having ever heard of breast self-examination (Pearson’s χ^2^ = 31.753, df = 5, *p* < 0.002). Although awareness was generally high across all age groups, the percentage of women who reported having heard of breast self-examination was lower among those aged 21–24 years (84.3%) and higher among women aged 55–64 years (97.3%) and 45–54 years (97.2%). In addition, educational level was significantly associated with awareness of breast self-examination (Pearson’s χ^2^ = 8.781, df = 3, *p* = 0.032). The proportion of women who had heard of breast self-examination increased with educational level, ranging from 87.5% among women with primary education to 97.5% among those with postgraduate education. These findings suggest that awareness of breast self-examination was higher among older and more highly educated women.

Linear regression analysis was performed to identify the significant sociodemographic and reproductive factors associated with the three subscales (Table 5).

Specifically, higher educational level (B = 2.733, β = 0.089, *p* = 0.022) and higher monthly family income (B = 2.868, β = 0.136, *p* = 0.001) were associated with higher attitude scores. In contrast, place of residence was negatively associated with attitude (B = −6.066, β = −0.107, *p* = 0.005). Women living in rural areas had significantly lower attitude scores. The final model was statistically significant (F = 9.791, *p* < 0.001) but explained only 5.5% of the variance in attitudes (R^2^ = 0.055, adjusted R^2^ = 0.049), which means weak explanatory.

Concerning the subscales, knowledge and perceptions about breast cancer, educational level, place of residence, monthly family income, and history of normal childbirth were identified as significant predictors. More specifically, higher educational level (B = 2.819, β = 0.103, *p* = 0.010) and higher monthly family income (B = 2.084, β = 0.110, *p* = 0.004) were associated with higher knowledge scores. Place of residence showed a negative association with knowledge (B = −4.282, β = −0.084, *p* = 0.026). Women residing in rural areas were more likely to report lower knowledge scores. The history of vaginal delivery was also negatively associated with knowledge (B = −2.577, β = −0.126, *p* = 0.001). The final model was statistically significant (F = 10.899, *p* < 0.001) but explained only 6.0% of the variance in knowledge (R^2^ = 0.060, adjusted R^2^ = 0.055), which means weak explanatory.

For subscale barriers to mammographic screening, the regression model identified monthly family income as the only statistically significant predictor. Specifically, higher monthly family income was associated with lower barrier scores (B = −1.108, β = −0.080, *p* = 0.039). The final model was statistically significant (F = 4.262, *p* = 0.039), although it explained a very small proportion of the variance in barriers (R^2^ = 0.006, adjusted R^2^ = 0.005).

The regression models identified several statistically significant factors associated with the Breast Cancer Screening Beliefs subscales. However, the standardized beta coefficients of all models were small, indicating weak associations. Therefore, the findings should be interpreted as statistically significant but limited in explanatory strength.

## 4. Discussion

The results of this study provide a multidimensional picture of women’s attitudes, knowledge, and practices toward breast cancer screening. Overall, the sample was characterized by positive attitudes and high awareness of prevention methods, but this knowledge did not translate into the systematic adoption of preventive behaviors. The high levels of awareness observed in this study may partly reflect the demographic characteristics of the sample, which included a large proportion of highly educated, employed, and urban-dwelling women, groups that have previously been associated with greater health literacy and higher engagement in preventive healthcare. This finding is consistent with recent systematic reviews, which suggest that knowledge alone is not sufficient to promote participation in screening and sustained preventive behaviors [3,4].

### 4.1. High Awareness, but No Corresponding Systematic Prevention

Awareness of basic prevention methods was particularly high, with almost all participants reporting knowledge of breast self-examination, clinical examination, and mammography. However, when considering practical implementation, significant gaps emerge. Although the majority had heard of self-examination, a smaller percentage knew how to perform it correctly, and even fewer women performed it regularly. This finding suggests a gap between knowledge and practical ability. A similar picture was observed in clinical breast examination, where, despite satisfactory recent participation, there was also a group that had never been examined. Regarding mammography, although it was almost universally known, its systematic application remained limited, with a significant percentage of women being examined less frequently or having never undergone it. However, this finding should be interpreted with caution, as the study included a considerable proportion of younger women who may not yet have been eligible for routine mammography screening according to national recommendations. Therefore, the proportion of women reporting no previous mammography should not be interpreted exclusively as non-adherence to screening guidelines. Consequently, acceptance of the importance of preventive examinations does not seem to be accompanied by a correspondingly stable participation in screening. This finding is in line with international literature, where knowledge often outweighs consistent preventive behavior [8,9,17]. Similar findings have been recorded in Greece. Studies have shown that, despite the existence of basic information, regular self-examination remains limited and knowledge is often fragmentary [18,19]. Furthermore, even in populations with higher health literacy, such as health professionals, the systematic implementation of self-examination is not given [20]. Internationally, the knowledge-practice gap has been associated with multifactorial mechanisms, including low self-efficacy, fear of diagnosis, lack of practical skills, and barriers to access. However, these factors were not directly assessed in the present study and should therefore be considered as potential explanations suggested by the existing literature [7,21]. Furthermore, it has been shown that knowledge without training and behavioral reinforcement remains superficial [10]. Therefore, the findings of the present study reinforce the need for interventions that go beyond simple information.

### 4.2. Age and Differentiation of Attitudes and Knowledge

Of particular interest is the effect of sociodemographic characteristics. Age, educational level, place of residence, and family income significantly differentiated both attitudes and knowledge levels. Women aged 31–40 years showed more positive attitudes towards general health checks, while women aged 21–30 years showed higher levels of knowledge. In contrast, older age groups recorded lower performance, which may reflect differences in access to information and the relationship with prevention. Age emerged as an important differentiating factor. Middle-aged women showed more positive attitudes, while younger women showed higher levels of knowledge. This finding is consistent with international studies showing that younger women have greater exposure to information, but not necessarily greater participation in screening [22]. The literature suggests that age influences behavior through factors such as perceived vulnerability and contact with the health system, which explains the conflicting findings in the international literature [4].

### 4.3. Education and Income as Social Determinants

Educational level and family income also acted as clear differentiating factors, as women with higher education and higher income showed more favorable attitudes and more knowledge. Similarly, women living in urban areas performed better than those living in rural areas, highlighting the importance of social and geographical inequalities in access to prevention and information. Multivariate analysis confirmed that education and income were positive predictors of both attitudes and knowledge, whereas rural residence was negatively associated with these dimensions. Although the models were statistically significant, they explained only a small proportion of the total variance, suggesting that other factors, such as psychological variables, health experiences, or the level of health literacy, may also be involved. Specifically, educational level and income were strong predictors, a finding confirmed in both Greek and international studies [3,5,6]. Women of higher socioeconomic status are more likely to participate in screening, which is associated with increased health literacy and better access to services. The systematic review by Georga et al. (2025) [4] confirms that education is one of the most important factors in shaping attitudes and knowledge about breast cancer.

### 4.4. Place of Residence and Access Inequalities

Place of residence also emerged as important, with women in urban areas performing better. This finding is supported by studies showing that women in rural areas face more barriers, such as geographical isolation and limited access to health services [13,23]. A Qualitative study in Greece has shown that rural women often delay screening due to practical and psychological barriers [12]. At the same time, the development of organized screening programs, such as the “Fofi Gennimata” program, attempts to reduce these inequalities through universal access [11].

### 4.5. Reproductive History and Social and Psychosocial Factors

Regarding reproductive history, some indicators, such as number of children, number of pregnancies, and history of normal birth, were mainly associated with knowledge level. Women without children or without previous pregnancies showed higher knowledge, a finding that may be related to less family burden and greater ability to deal with personal health issues. The findings regarding reproductive history indicate that women with fewer family obligations display higher levels of knowledge. This result can be interpreted through social rather than biological factors, as the increased burden of family roles limits the ability to engage in prevention [9]. Despite the importance of demographic characteristics, the models explained only a small proportion of the variance, suggesting that additional factors, including psychosocial determinants, may contribute to screening behaviors. However, these variables were not measured in the present study, and their role remains speculative. The international literature highlights the role of self-efficacy, health beliefs, and fear as important determinants of screening participation. Nevertheless, these variables were not measured in the present study and therefore cannot explain the observed findings directly [7,15].

As far as the regression analyses of breast cancer screening beliefs are concerned, several predictors were statistically significant; however, the models explained only a low proportion of the variance. This suggests that the included sociodemographic and reproductive variables account for only a limited proportion of the variability in breast cancer screening beliefs. This finding is not unexpected, as screening-related beliefs and preventive health behaviors are complex and multifactorial. They may be influenced by additional psychosocial, cultural and health-system-related factors, such as perceived susceptibility, perceived benefits, perceived barriers, self-efficacy, previous experiences with healthcare services and physician recommendation, which were not fully captured in the present regression models.

### 4.6. Limitations

This study has some limitations that should be considered when interpreting the results. First, its cross-sectional design does not allow for causal inferences and only for the identification of associations between variables. Furthermore, data collection was based on self-reported responses, which may introduce bias or recall bias. At the same time, the sample may not be fully representative of the general population, limiting the generalizability of the results. The use of a non-probability snowball sampling strategy and predominantly digital recruitment may have favored the participation of women who were more health-conscious, more socially connected, and more engaged with digital technologies, resulting in the overrepresentation of women with higher educational levels, urban residence, and greater digital literacy. Consequently, the findings should be interpreted with caution and cannot be considered fully representative of the entire female population of Greece. Women with limited digital literacy or restricted access to online technologies may have been less likely to participate and, therefore, may be underrepresented in the present sample. In addition, the study focused primarily on sociodemographic and reproductive factors and did not directly assess important psychosocial determinants of screening behavior, including perceived risk, fear of diagnosis, self-efficacy, trust in health services, health literacy, previous abnormal findings, and physician recommendation. In addition, healthcare system factors, such as accessibility of services and practical barriers to screening, were not comprehensively evaluated. Future studies should incorporate these variables to provide a more comprehensive understanding of breast cancer screening behaviors. Although several reproductive variables were examined because they may reflect women’s interactions with healthcare services and preventive behaviors, the study did not include more direct measures of access to screening services, such as physician recommendation, previous screening experiences, or structural barriers to healthcare access.

## 5. Conclusions

In summary, the findings of this study highlight that women have largely positive attitudes and a high level of information regarding breast cancer screening, but this knowledge does not translate into consistent and systematic preventive behavior. The main conclusion concerns the existence of a clear gap between knowledge and practice, which appears to be a critical obstacle to the effective implementation of prevention. At the same time, the important role of sociodemographic factors, such as education, income, and place of residence, is highlighted, which substantially influence both the level of knowledge and attitudes towards preventive screening. Women of lower socioeconomic status and those residing in rural areas appear to be at a greater disadvantage in terms of access to and participation in prevention. In addition, age and reproductive history appear to be associated with differences in cognition and behavior, reflecting broader social and functional parameters. At the same time, the low percentage of variance explained by demographic models suggests that preventive behavior is also influenced by psychosocial factors, such as self-efficacy, health beliefs, and fear. Therefore, promoting prevention cannot be limited to providing information alone, but requires comprehensive interventions that strengthen skills, reduce barriers, and consider social inequalities. Finally, the study findings can be used to design targeted public health policies, aiming to improve participation in screening and reduce inequalities in breast cancer prevention.

## Figures and Tables

**Table 1:** Demographic characteristics of the sample.

	Frequency	Percent
Age group		
21–24	140	11.4
25–34	238	19.3
35–44	289	23.4
45–54	322	26.1
55–64	188	15.3
65+	56	4.5
Marital status		
Married	784	63.9
Divorced/Separated	93	7.6
Single	297	24.2
Widow	52	4.2
Educational level		
Primary education	26	2.1
Secondary education	308	25.1
Tertiary education	603	49.2
Postgraduate studies	289	23.6
Place of residence		
Urban area	948	76.9
Rural area	285	23.1
Occupational status		
Employed	898	72.8
Unemployed	264	21.4
Retired	71	5.8
Monthly Family Income		
Up to €500	59	4.9
€501–€700	102	8.4
€701–€1000	258	21.3
€1001–€1500	352	29.0
>€1501	442	36.4

**Table 2:** Reproductive history.

	Frequency	Percent
Number of children		
None	289	26.7
One	225	20.8
Two	445	41.1
More Than 2	125	11.5
Number of pregnancies		
None	277	25.7
One	184	17.1
Two	351	32.6
More Than 2	264	24.5
Normal childbirth		
None	691	59.8
One	146	12.6
Two	229	19.8
More Than 2	89	7.7
Caesarea		
None	766	66.6
One	171	14.9
Two	183	15.9
More Than 2	30	2.6
Number of miscarriages/abortions		
None	443	56.8
One	195	25.0
Two	100	12.8
More Than 2	42	5.4
IVF history		
yes	92	7.5
Number of IVF		
One	35	38
Two	26	28.3
More Than 2	31	33.7

**Table 3:** Breast screening practices.

	Frequency	Percent
Family history of breast cancer		
No	916	78.5
Yes	234	20.1
I don’t know/I don’t answer	17	1.5
Friends’ history of breast cancer		
No	654	56.1
Yes	490	42.1
I don’t know/I don’t answer	21	1.8
Awareness of breast self-examination		
Yes	1125	96.5
Knowledge about how to perform breast self-examination		
Yes	922	79.5
Frequency of breast self-examination		
Never	223	19.2
Once a year	256	22.0
Once every few months	418	36.0
At least once a month	264	22.7
Knowledge about clinical breast examination		
Yes	1091	94.5
Last clinical breast examination		
A year or less	571	49.4
More than a year & less than two years	244	21.1
Two to three years	104	9
More than three years	83	7.2
Never had one	154	13.3
Reason for last clinical breast exam visit		
Due to a chest problem	49	5.0
My doctor suggested it as part of a health check-up	377	38.8
I decided to do it as part of a health check-up	546	56.2
Knowledge about mammogram		
Yes	1093	95.0
Frequency of mammogram (only for women 45+ years old)		
Once every two years	487	86
Once every three years and more	69	12.2
Never had one	10	1.8
Preference for a mammography operator		
No preference	686	60.2
Men	10	0.9
Women	444	38.9
Friends’ mammography uptake		
No	244	21.2
Yes	508	44.2
I don’t know	397	34.6

**Table 4:** Significant differences between sociodemographic and reproductive characteristics and Breast Cancer Screening Beliefs.

Factors	Attitudes Towards General Health Check-Ups	Knowledge and Perceptions About Breast Cancer	Barriers to Mammographic Screening	Awareness of Breast Self-Examination	
	Mean (S.D)	Mean (S.D)	Mean (S.D)	No	Yes
				% (n)	% (n)
Age group					
21–24	78.2 (21.6)	84.5 (16.3)		15.7% (22)	84.3% (118)
25–34	83.2 (22.4)	79.2 (23.8)		3.36% (8)	96.6% (230)
35–44	81.2 (22.7)	79.4 (22.1)		3.4% (10)	96.5% (279)
45–54	82.8 (21.9)	77.8 (20.8)		2.8% (9)	97.2% (313)
55–64	74.2 (28.7)	72.3 (25.8)		2.7% (5)	97.3% (183)
65+	68.8 (20.8)	70.4 (23.7)		5.4% (3)	94.6% (53)
*p* value	0.007	0.003		0.002	
Educational level					
Primary education	57.0 (35.2)	60.2 (25.5)		12.5% (3)	87.5% (21)
Secondary education	77.4 (25.7)	74.9 (23.8)		5.0% (14)	95.0% (268)
Tertiary education	79.8 (23.0)	80.7 (20.1)		3.0% (17)	97.0% (557)
Postgraduate studies	83.4 (19.4)	82.2 (18.7)		2.5% (7)	97.5% (273)
*p* value	0.001	0.001		0.032	
Place residence					
Urban area	80.9 (22.8)	80.4 (20.5)	89.9 (15.4)		
Rural area	75.2 (25.3)	75.3(23.0)	87.7 (15.5)		
*p* value	0.001	0.001	0.043		
Family income					
Up to €500	77.1 (22.8)	77.2 (19.3			
€501–€700	70.3 (30.8)	74.2(23.8)			
€701–€1000	78.0 (23.9)	76.3 (24.4)			
€1001–€1500	78.2 (22.7)	79.9 (18.6)			
>€1501	84.5 (20.7)	81.8 (20.0)			
*p* value	0.001	0.002			
Children					
none	77.8 (23.0)	82.2 (17.8)			
one	80.3 (23.3)	79.2 (22.6)			
two	81.5 (23.1)	77.3 (22.0)			
more than two	75.5 (26.3)	75.8 (24.3)			
*p* value	0.045	0.010			
Pregnancy history					
none		81.7 (17.6)			
one		79.6 (22.9)			
two		78.5 (21.1)			
more than two		75.7 (23.8)			
*p* value		0.016			
Vaginal delivery					
none		81.4 (18.5)			
one		77.5 (24.2)			
two		74.7 (23.9)			
more than two		72.5 (26.5)			
*p* value		0.001			

**Table 5:** Linear regression analysis of Breast Cancer Screening Beliefs Subscale.

	Unstandardized Coefficients		Standardized Coefficients	Sig.	95.0% Confidence Interval for B	
	B	Std. Error	Beta		Lower Bound	Upper Bound
Dependent variable: Attitudes toward general health check-ups						
(Constant)	68.626	5.294		0.000	58.231	79.021
Educational level	2.733	1.192	0.089	0.022	0.393	5.074
Place of residence	−6.066	2.153	−0.107	0.005	−10.293	−1.839
Monthly family income	2.868	0.820	0.136	0.001	1.258	4.478
Dependent variable: Knowledge and perceptions about breast cancer						
(Constant)	69.741	4.798		0.000	60.321	79.161
Educational level	2.819	1.088	0.103	0.010	0.683	4.955
Place of residence	−4.282	1.924	−0.084	0.026	−8.060	−0.505
Monthly family income	2.084	0.729	0.110	0.004	0.653	3.514
Normal childbirth	−2.577	0.781	−0.126	0.001	−4.110	−1.044
Dependent variable: Barriers to mammographic screening						
(Constant)	15.057	2.184		0.000	10.769	19.345
Monthly family income	−1.108	0.537	−0.080	0.039	−2.162	−0.054

## Data Availability

The data presented in this study are available on request from the corresponding author due to ethical reasons.

## Abbreviations

The following abbreviations are used in this manuscript:

BCSBQ	Breast Cancer Screening Beliefs Questionnaire
